# Long-Term Single Cell Analysis of *S. pombe* on a Microfluidic Microchemostat Array

**DOI:** 10.1371/journal.pone.0093466

**Published:** 2014-04-07

**Authors:** Jean-Bernard Nobs, Sebastian J. Maerkl

**Affiliations:** Institute of Bioengineering, School of Engineering, Ecole Polytechnique Federale de Lausanne, Lausanne, Switzerland; Texas A&M University, United States of America

## Abstract

Although *Schyzosaccharomyces pombe* is one of the principal model organisms for studying the cell cycle, surprisingly few methods have characterized *S. pombe* growth on the single cell level, and no methods exist capable of analyzing thousands of cells and tens of thousands of cell division events. We developed an automated microfluidic platform permitting *S. pombe* to be grown on-chip for several days under defined and changeable conditions. We developed an image processing pipeline to extract and quantitate several physiological parameters including cell length, time to division, and elongation rate without requiring synchronization of the culture. Over a period of 50 hours our platform analyzed over 100000 cell division events and reconstructed single cell lineages up to 10 generations in length. We characterized cell lengths and division times in a temperature shift experiment in which cells were initially grown at 30°C and transitioned to 25°C. Although cell length was identical at both temperatures at steady-state, we observed transient changes in cell length if the temperature shift took place during a critical phase of the cell cycle. We further show that cells born with normal length do divide over a wide range of cell lengths and that cell length appears to be controlled in the second generation, were large newly born cells have a tendency to divide more rapidly and thus at a normalized cell size. The platform is thus applicable to measure fine-details in cell cycle dynamics, should be a useful tool to decipher the molecular mechanism underlying size homeostasis, and will be generally applicable to study processes on the single cell level that require large numbers of precision measurements and single cell lineages.

## Introduction

Cells have been historically studied with population-level measurements, on the assumption that an individual cell’s phenotype is well-described by the population average. However, recent studies have shown that considerable variations in mRNA levels, protein levels, doubling time, and size exist between isogenic cells [1]–[5]. Thus clonal cultures exhibit large variation on the single cell level. This variation raises questions related to how robustness can be achieved, information is processed and transmitted over several cell-cycles. Tools to measure single cell variability are becoming available [6], [7], but generally lack both throughput and precision.

Several approaches exist for measuring growth of microbial cells [8]. Optical density measurements estimate the cell number in liquid medium over time from which the doubling time can be estimated. Competition assays are used to analyze small differences in growth rates. Here different strains or clones are pooled and grown in liquid culture, with each strain or clone carrying a unique marker. The relative abundance of each strain/clone can then be determined using next generation sequencing [9], microarray analysis [10], [11], FACS [12], or plating [13], from which the relative fitness for each strain/clone can be determined.

All of these approaches are population level measurements and thus do not allow assessment of single cell parameters. A second drawback is the fact that neither morphological nor phenotypic changes over a single lineage can be assessed. Although the environment can be perturbed in these assays, transient changes are not feasible. A method capable of following thousands of continuously dividing cells over extended periods of time would be extremely useful for deriving accurate growth rate measurements, and to link single cell phenotypes with cell cycle.

Several attempts have been made to develop tools for single-cell measurements. One simple tool to measure physiological parameters of single microbial cells is the agar pad [14], were cells are placed between a coverslip and a thin piece of agar. This is a straightforward technique but also has several limitations: i) observation time is limited as cells are not removed and ii) dynamic culturing such as medium changes can’t be conducted.

Microfluidic devices allow for the precise handling of fluids, and thus have become popular approaches for conducting single cell studies [6]. In the first implementations, cells were grown sandwiched between a cellulose membrane and a coverslip with microfluidic channels located above the membrane to control medium flow and allowing for medium exchange [15], [16]. In integrated microfluidic platforms, cells were grown in small traps or confined regions where they could be supplied with nutrients from nearby channels by diffusion [5], [6], [17], [18] and some of those tools were used to grow *S. pombe*. Commercial microfluidic approaches are also available such as the yeast Onyx of CellAsic product. This setup was used to monitor growth of *S. pombe* in a nutrient limited environment and several parameters, such as doubling time and symmetry of division were extracted [19]. Nonetheless, all of these approaches still face the issue that cells can only be cultured for limited duration and cell tracking is challenging.

A different approach is to trap individual cells while daughter cells are removed, which permits long-term tracking of a few cells over many generations. Such approaches were developed for *Saccharomyces cerevisiae* [20]–[22] and *Escherichia coli* [23], [24]. However, these approaches only return lineages for the cells initially trapped. Furthermore, the design of the devices in these experiments limits the number of cells that can be imaged at a time [24].

We have developed a microfluidic system that alleviates these issues. We show here that our microfluidic platform can quantitate over 100000 divisions in a single experiment, track cells over multiple generations, and reconstruct lineages. *S. pombe* can be cultured on-chip for as long as a week, in a controlled environment which can be rapidly changed (temperature shifts, or medium switches). Furthermore, no specific marker is required for cell segmentation or tracking. Cells are introduced by simple flow loading, devices are readily available from microfluidic foundries (Caltech, Stanford), and device schematics and software tools are available at cellbase.epfl.ch.

## Results

### Microfluidic Device and Image Processing

**Figure 1:**
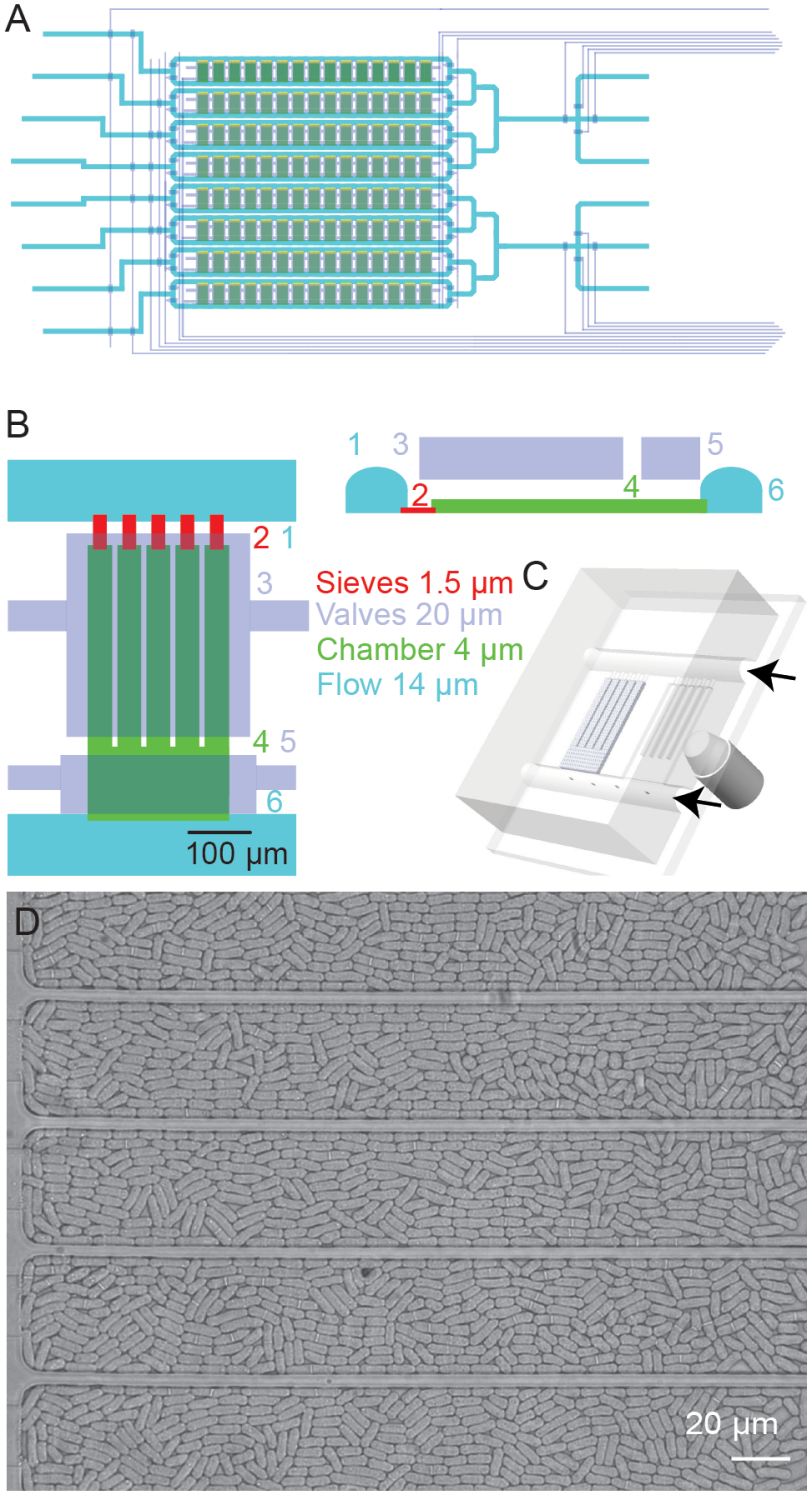
Microfluidic device design. (A) Schematic of the *S. pombe* microchemostat array. Control layer channels are shown in grey, while flow channels are shown in green and cyan. The device consists of 120 microchemostats divided into 8 individually addressable rows. (B–C) A detailed schematic of a single microchemostat chamber. Each growth chamber (4) is supplied with medium through two flow channels (1,6). Sieve channels (2) allow molecules the perfuse the growth chamber while preventing cell escape. Each growth chamber is controlled by a chamber valve (5) and a button membrane (3). (D) A bright-field image of one growth chamber filled to confluence with *S. pombe* cells.

**Figure 2:**
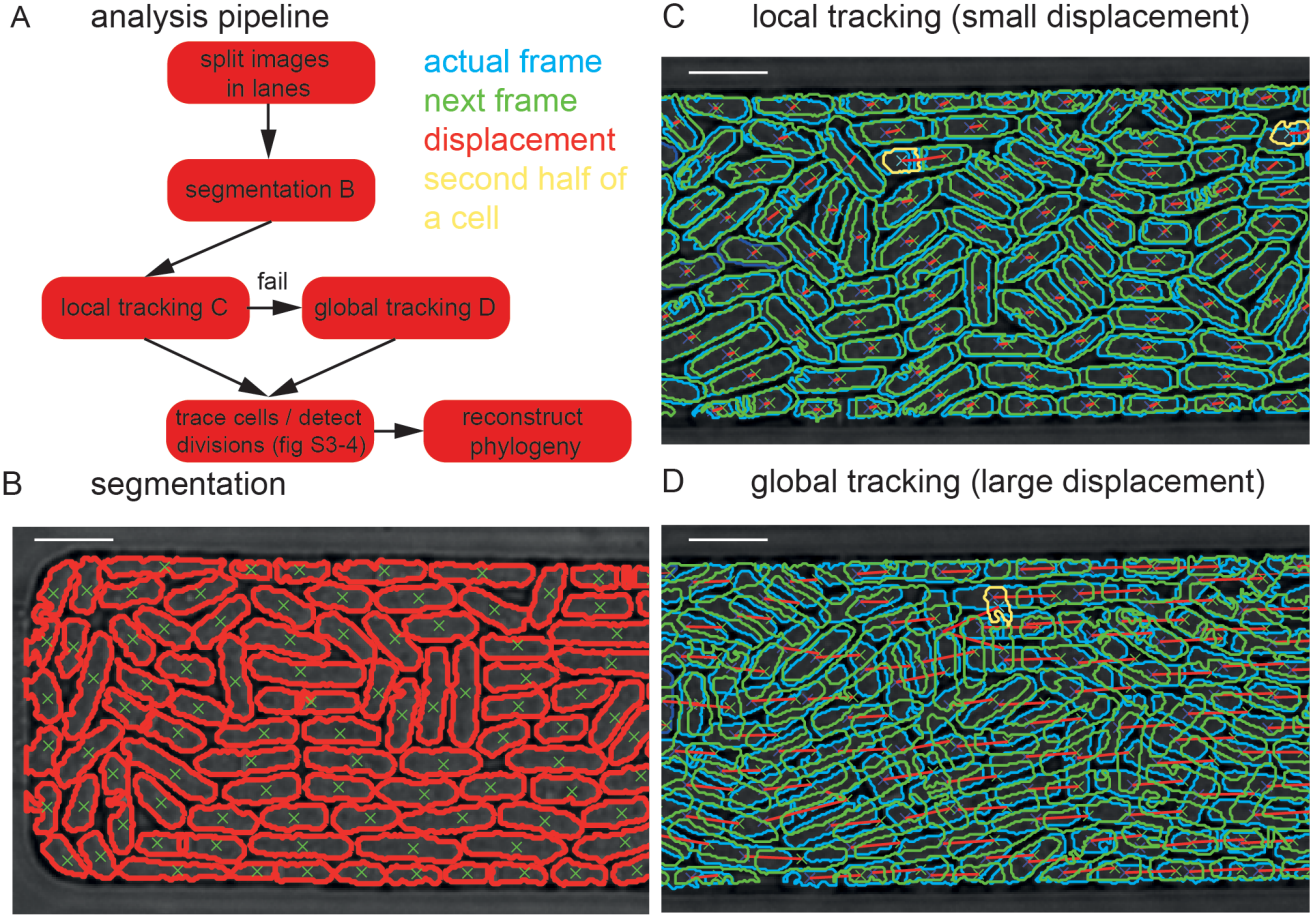
Image processing pipeline and cell tracking. (A) A flow diagram showing all analysis steps of the image processing pipeline. First, each growth chamber is divided into lanes as defined by the microfluidic ‘highway’ structures. Next, individual cells are segmented (B) and tracked using either a local (C) or a global (D) tracker. All scale bars are 10 μm.

We designed a microfluidic device consisting of 120 microchemostat growth chambers based on a design previously applied to large-scale single cell studies in *S. cerevisiae* (Fig. 1) [25]. The chip is fabricated using standard multilayer soft lithography [26]. Each microchemostat chamber contains five ‘highways’ which help guide the cells to the outlet of each chamber and facilitates cell segmentation and tracking. The device is placed on an automated microscope enclosed in a temperature controlled environment. Cells are flow loaded on the device and can be grown for long periods of time. The longest duration of an experiment to-date was 1 week. Each of the 120 microchemostats can be imaged every 2.5 minutes. In the experiments described here, 50 microchemostats per device were imaged every minute to improve the temporal resolution and consequently enhance the accuracy of our cell-tracking algorithm (Fig. 2, Movie S1). Details on the device and image processing algorithms are given in the methods section.

### Platform Performance

On-chip cell growth rate was slightly slower compared to bulk culture, with the average on-chip doubling time of 128±2 minutes being 10% higher than that measured for a standard liquid culture (115±2 minutes). These differences are comparable to differences previously observed for *S. cerevisiae* with various microfluidic devices.

**Figure 3:**
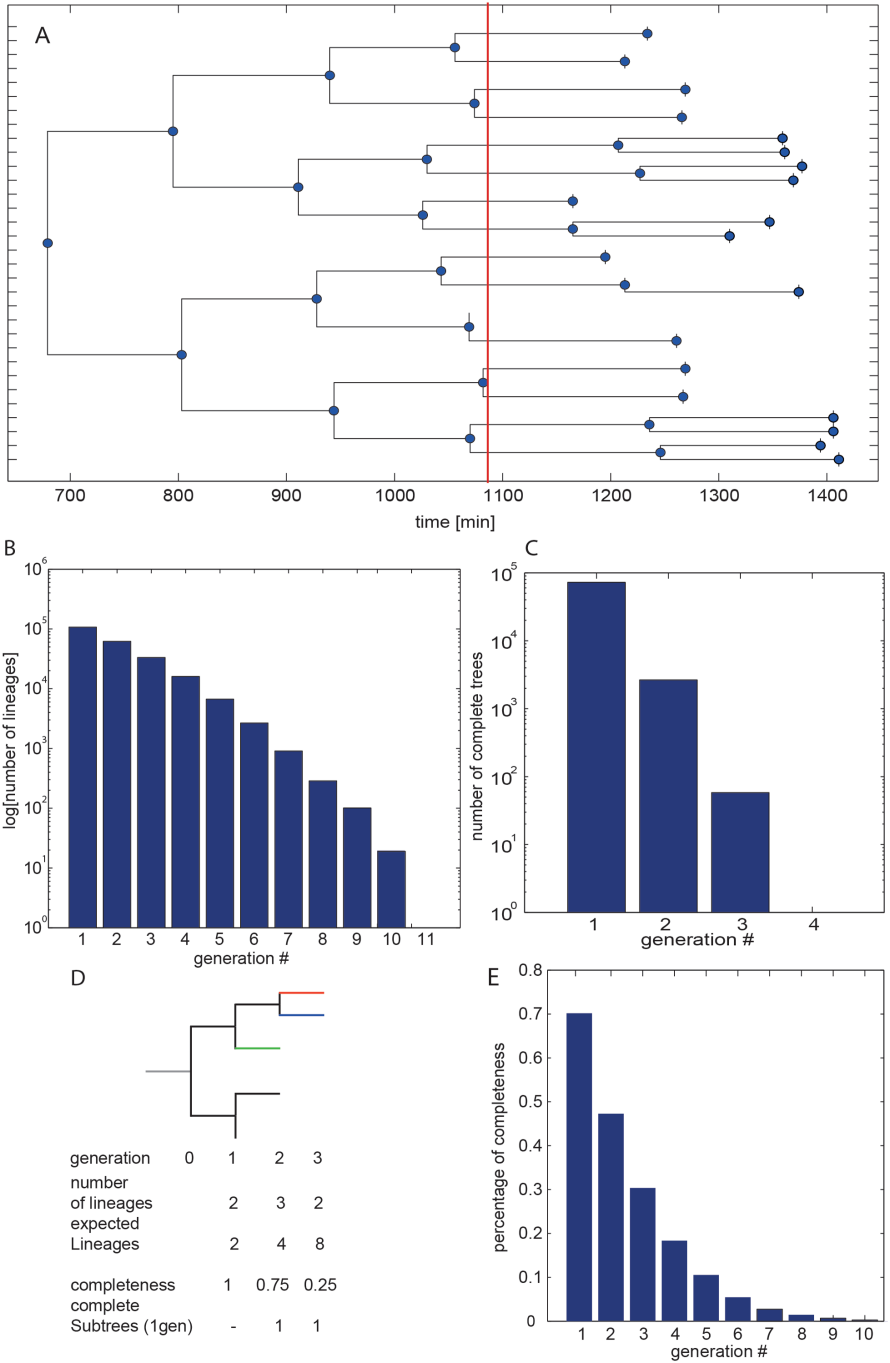
Large-scale lineage tracking. (A) A five generation lineage tree reconstructed by our image processing pipeline. (B) The number of lineages that could be traced over a given number of generations during a single experiment covering 50 hours of cell growth. (C) The number of complete lineage trees 1, 2, or 3 generations in length. (D) A schematic describing our definition of percent completeness of a lineage tree and the corresponding average completeness of lineage trees of a given number of generations in length (E).

The primary objective for our microfluidic platform was to track thousands of cells through at least one complete cell division cycle to obtain precise growth rate measurements. A secondary objective was to track cells over several generations to construct lineage trees. Analyzing micro-colonies is the most effective way of generating complete lineage trees [17], whereas devices that immobilize single cells are most effective for long term monitoring of cells over many generations [21]. In our approach we observe a continuously growing micro-colony over long time-periods, conceptually placing our approach between observing a micro-colony and cell trapping approaches. To assess the performance of our platform in terms of constructing single-cell lineages we quantitated the number of lineage trees generated, their size, and completeness. Figure 3A shows a large lineage tree, encompassing 5 generations, reconstructed from our tracking data for one device running for 50 hours. To quantify the effectiveness of our platform in cell tracking and lineage tree generation we defined several metrics. First, we assessed how many cells could be tracked for a given number of generations. We could track 107103 cells over one generation, or one complete cell cycle (Fig. 3B). We were able to track 19 lineages encompassing 10 generations and 95 lineages 9 generations in length (Fig. 3B). By comparison, current cell-trapping based approaches generally track ∼50 *S. cerevisiae* cells for up to 60 generations in length [21], whereas micro-colony approaches can generate lineages up to 8 generations in length [27].

A second metric useful in describing the performance of a cell-tracking platform is the number of complete trees and the completeness of trees of a given number of generations. Obtaining complete lineage trees is challenging, even for micro-colony approaches. The primary reason is the accuracy of the segmentation and tracking algorithms. We were able to obtain 58 complete trees 3 generations in length, and 2623 complete trees encompassing 2 generations (Fig. 3C). We define lineage tree completeness as the number of observed lineage leaves divided by the expected number of leaves for a tree of a given length. The example given in Figure 3D shows an incomplete lineage tree, which is 25% complete for a 3 generation tree and 75% complete for a 2 generation tree. On average we achieved 70% completeness for trees 1 generation in length and 47% completeness for trees 2 generations in length (Fig. 3E).

### Large-scale Single Cell Growth Analysis

**Figure 4:**
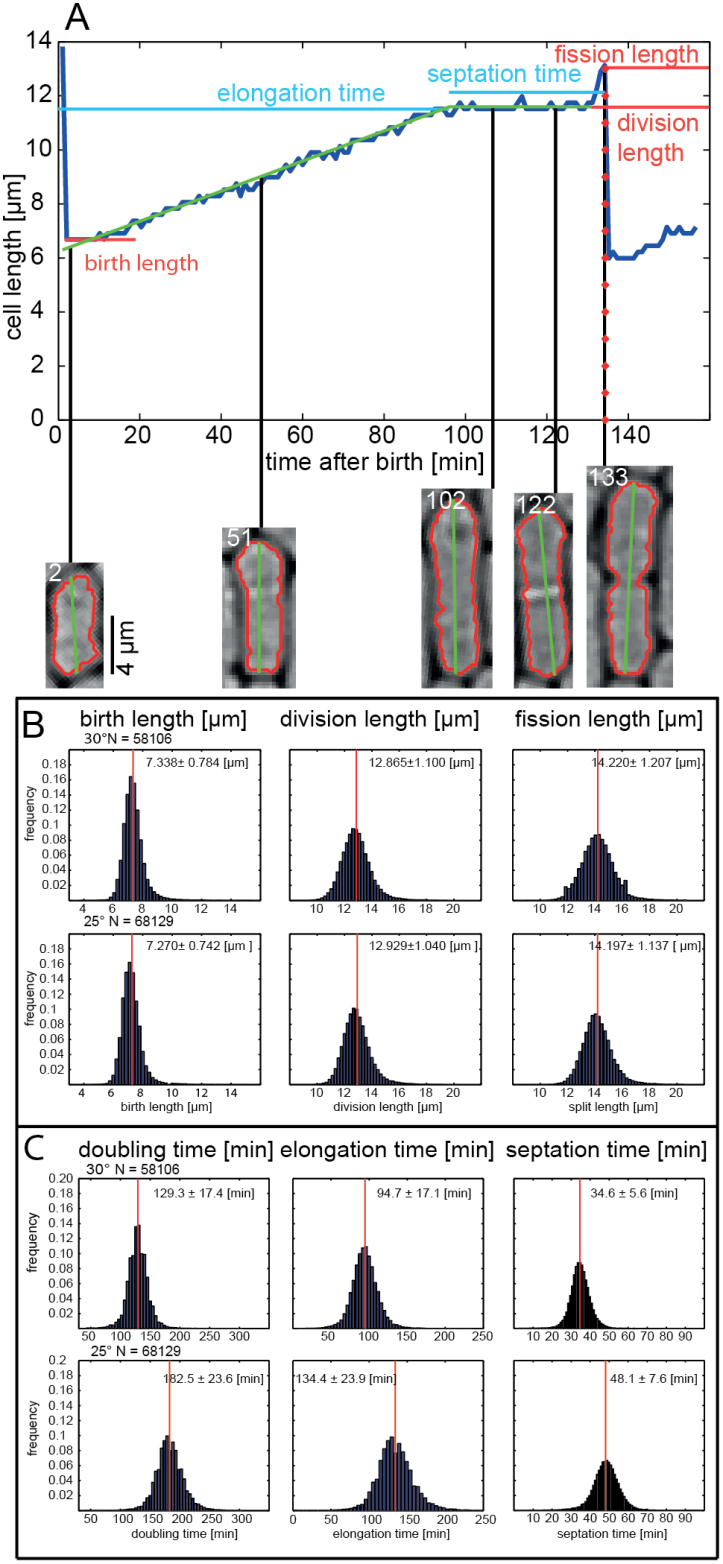
Single-cell growth characterization. (A) Example of a single cell growth process and definition of the various parameters the image processing pipeline returns including: birth length, division length, fission length, elongation time, septation time, and elongation rate. Histograms of (B) single cell birth, division, and fission lengths and (C) doubling, elongation, and septation times before and after a temperature shift from 30°C to 25°C.

Figure 4 illustrates an entire single cell growth process and the various growth parameters we defined using cell-length and the cell septation profile as measured with a Mexican hat filter (Fig. S1). We defined two size-dependent parameters: (i) the birth length of a cell after fission and (ii) the division length, the length of a cell at the end of its elongation phase. Associated with the latter parameter is the fission length, which is the result of a rapid length increase after septation, seen by the sharp peak in the length profile prior to division (Fig. S2). This reshaping explains why the size at birth is not half of the size at division but rather half the fission length. We also defined three time-dependent parameters: (i) the elongation time, (ii) the septation time, and (iii) the doubling time, which is the sum of the elongation and septation times and equivalent to the doubling time conventionally measured. We also defined the elongation rate as the change in cell-length per unit time.

We used these parameters to determine the effect of changes in temperature on cell growth and morphology. Cells were initially grown at 30°C and then subjected to a temperature decrease to 25°C (Fig. 4B,C). We observed a total of 58106 cell divisions at 30°C and 68129 cell divisions at 25°C. Steady state birth length (7.3 μm), division length (12.9 μm), and fission length (14.2 μm) remained constant upon a 5°C temperature shift [28] and so did the respective variances of the distributions (∼10%). Doubling time increased by 53.2 minutes from 129.3±17.4 minutes to 182.5±23.6 minutes. Both septation and elongation time increased proportionally by 142% and 139%, respectively. Of the entire cell division cycle, cells spend 73% of the time in elongation phase and 26% in a non-elongating phase encompassing septation. Indicating that whichever process (or processes) is limiting division rates is affected equally in these two phases of the cell division phase. An appealing hypothesis is that a single cellular process is limiting and required/active in both phases. Synthesis of cell-membrane components could be one such possible factor setting limits to the doubling time. Although the elongation and septation times change dramatically upon a 5°C temperature shift, their coefficient of variation remains remarkably constant at 18% and 16%, respectively.

### Temporal Analysis of Cell Division Parameters during a Temperature Shift

**Figure 5:**
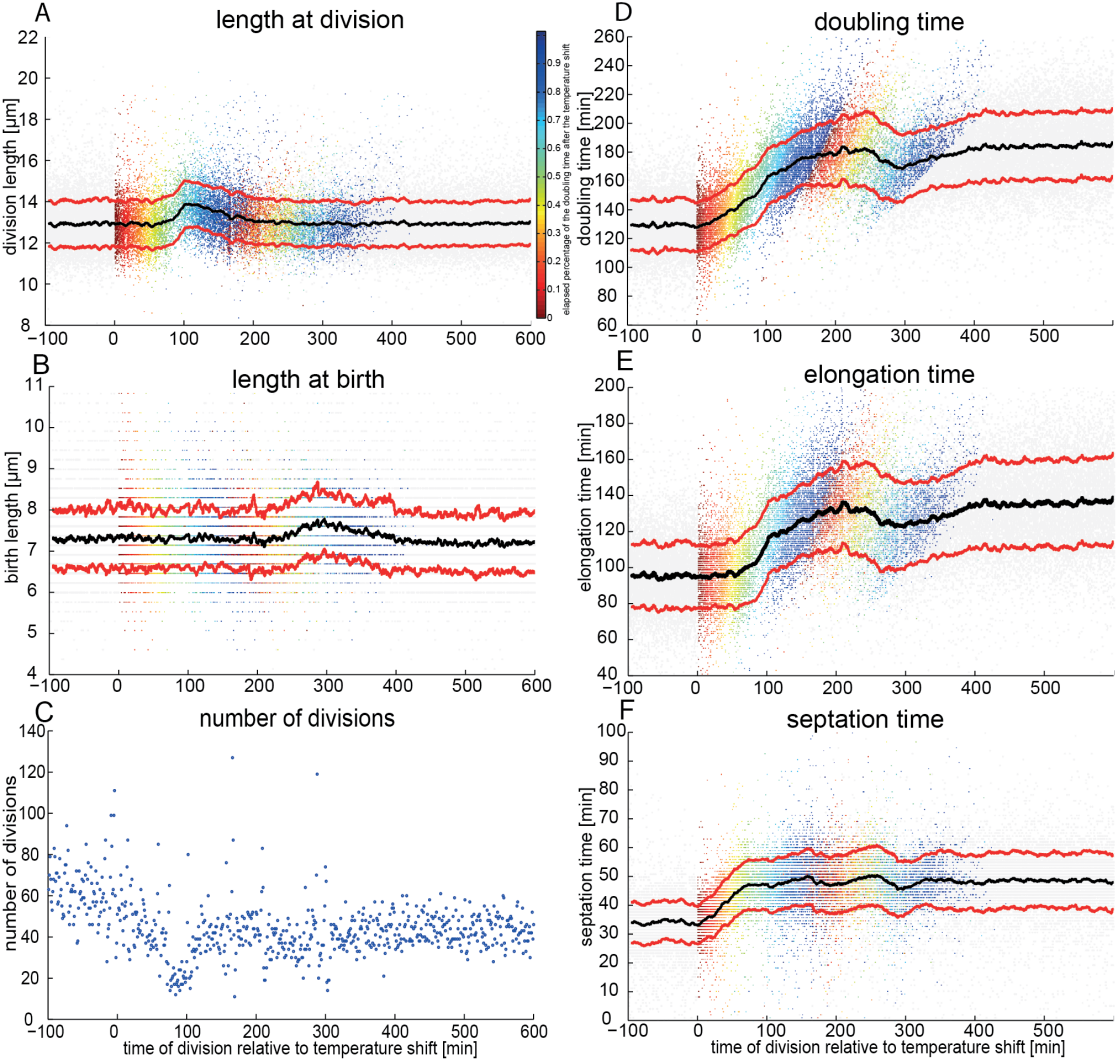
Various single cell parameters plotted over time and in respect to the temperature shift (0 min). Each data point is a single cell division event, and the color indicates the percentage of the division time that has elapsed after the temperature shift. The generation dividing during the temperature shift and the first generation after the temperature shift are shown. Black lines indicate the moving average and the red lines the standard deviation. (A) Division lengths, (B) birth lengths, (C) number of divisions per time bin, (D) doubling times, (E) elongation times, and (F) septation times are shown.

In the previous section we show the steady-state distributions of various parameters pre- and post-temperature shift. It is not known how cells that are exposed to a temperature shift during different phases of their cell cycle dynamically adjust these parameters. Since we observed a large population of unsynchronized cells, and our temperature shift occurs relatively quickly (with a transition (rise/fall) time of 40 minutes (80%) and 80 min (90%)), we could plot these parameters over time and in relation to the cell-cycle (Fig. 5).

Although cell size at birth and size at division are extremely robust as measured in steady-state cultures, we observed that these two parameters do in fact transiently vary when the temperature shift occurred at a specific time relative to the cell division cycle. Cell division length increased from the nominal length of 12.9 μm to 13.8 μm. Cells that spent up to 50% of their division cycle (the latter half of their cell-cycle) at the new, lower temperature divided at the nominal length. Cells that spent over 50% of their cell-cycle at the lower temperature divided at increasingly larger sizes, reaching a maximum of 13.8 μm if they spent 60% of their division cycle at the new temperature. If cells spent above 60% of their cell cycle at the new temperature, cell division length began to normalize until cells again divided at the nominal length if they spent their entire cell division cycle at the new temperature. This indicates that there is a temperature sensitive phase in the cell-cycle which does lead to abnormally long cells at division (around 60–70% into the cell cycle). Hence a temperature sensitive process that regulates cell-division length appears to be active during this part of the cell-cycle and possibly remains active till division. Those cells that divide at abnormally large cell-size, give rise to abnormally large daughter cells (Fig. 5B), but cell division length is consequently normal in the second generation. These results show that cell division is robust at steady-state, but is susceptible to temperature shifts if the shift occurs during a critical phase of the cell cycle and that these abnormal cell-division lengths are corrected in the next generation.

We could also discern time-dependent fine-structures in the doubling, elongation, and plateau times. As discussed previously these times increase proportionally upon a 5°C temperature shift. Doubling time begins to increase immediately upon temperature shift; cells that only spent a small fraction of their cell-cycle at the lower temperature already exhibit an increased doubling time. We also observed a pronounced and transient decrease in doubling time in the second generation after the temperature shift. This decrease in doubling time coincides with the slight increase in birth length also observed in the second generation after the temperature shift. This suggests that cells normalize larger birth length by decreasing the doubling time to consequently divide at the nominal length of 12.9 μm. This temporary decrease in doubling time arises primarily from a decrease in elongation time but not septation time (Fig. 5E–F). Elongation time showed an expected post-temperature shift lag until it increased. Interestingly the elongation time shows a biphasic behavior, rapidly increasing for cells that spent 40–60% of their cell-cycle at the new temperature, corresponding with the same critical window leading to drastic changes in division length identified above. After this initial and rapid change, elongation rate continued to increase to the steady-state elongation time, followed by the transient decrease mentioned above. The septation time on the other hand increased immediately for cells spending even a small fraction of their cell cycle at the new temperature, and increased to the steady-state septation time.

### *S. pombe* Length Homeostasis

**Figure 6:**
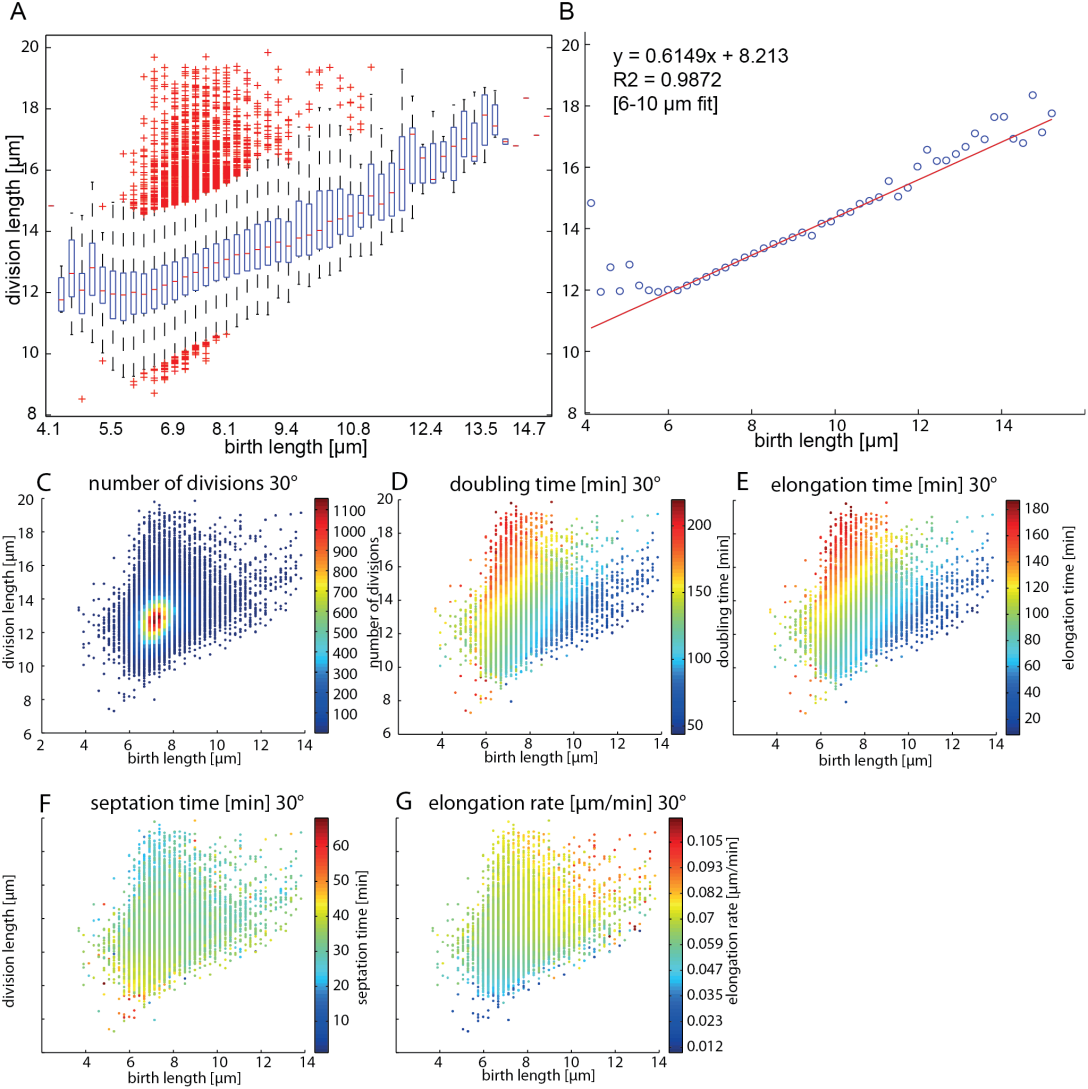
Cellular length homeostasis. (A) Box plots of single cell division lengths for cells with a given birth length. (B) The average values of the box plots shown on a scatter plot, with a linear regression fit to the data ranging from 6–10 μm birth lengths. (C) The raw data shown in (A–B). The color of each data point represents the number of single cell division per point. (D–G) Data as in (C) but each data point is now colored by (D) doubling time, (E) elongation time, (F) septation time, and (G) elongation rate.

**Figure 7:**
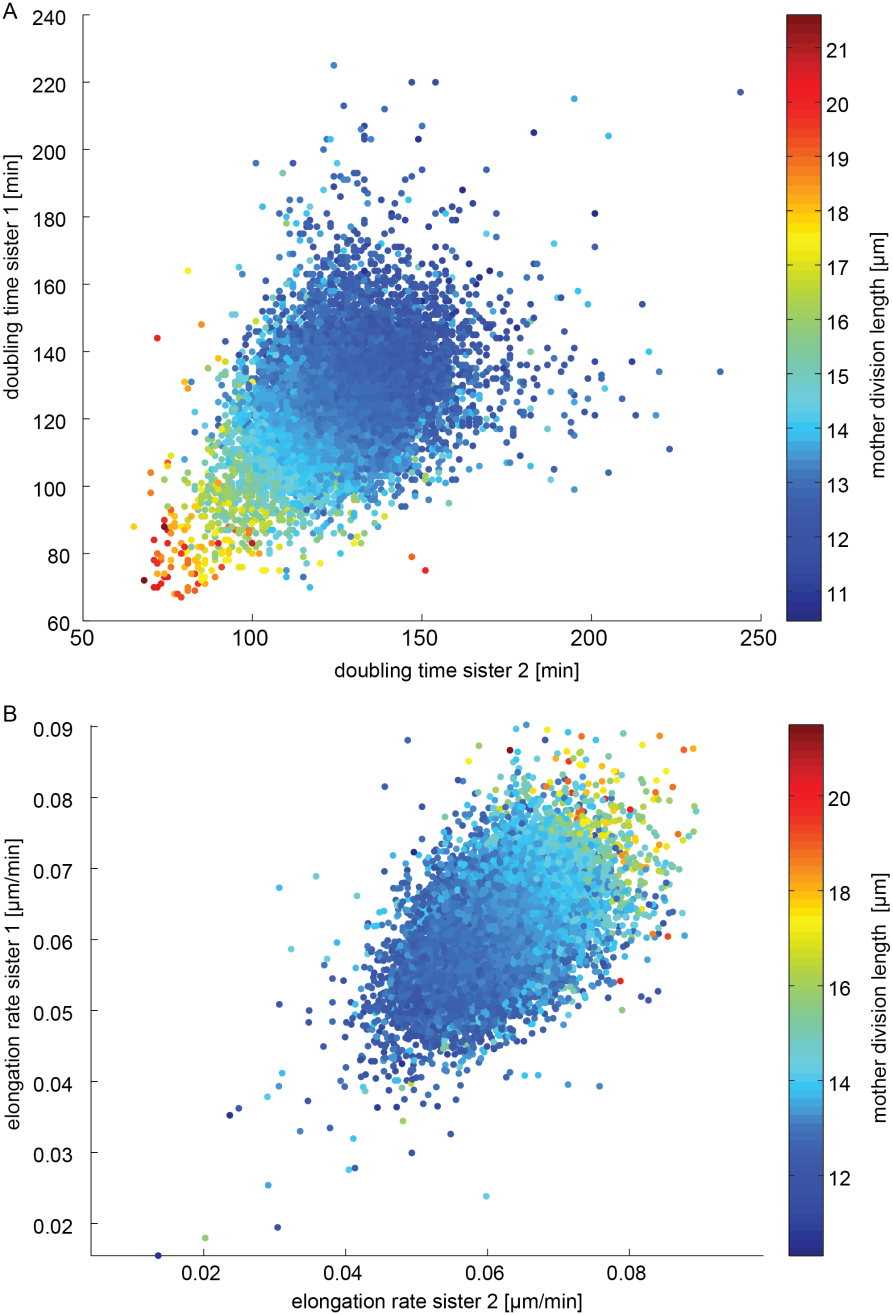
Comparison of sister cell (A) doubling times and (B) elongation rates. Sister pair data points are in both cases colored according to mother division length.

Cell length is well controlled in *S. pombe* and independent of temperature, while doubling times are dependent on temperature, presumably to achieve size homeostasis (Fig. 4). We also observed that, although size is homeostatic in steady-state conditions, it can vary if temperature shifts occur during critical phases of the cell cycle (Fig. 5), and that cells dividing at longer lengths give rise to large daughters, which then nonetheless appear to divide again at nominal lengths. We thus explored the relationship between birth and division length within a generation and across generations in more detail (Fig. 6,7).

Cells born with a normal cell length (∼7.3 μm) divide at lengths that span the entire range of observed cell sizes (Fig. 6A,C). Only very few cells are born with lengths below 6 μm. Most cells are born at a normal size and divide at a normal length, and birth length and division length are linearly correlated (Fig. 6B). We observed that the distribution averages of division lengths steadily decreases in respect to increasing birth lengths (the slope of the linear regression relating division length to birth length is 0.61). In other words, cells born with an abnormally large size have a tendency to divide at a relatively smaller division length. This leads to a normalization of cell-length and gives rise to size homeostasis. Size homeostasis is thus not a direct consequence of precise regulation of division lengths, but rather a consequence of regulating the division lengths of cells that are born with abnormally large sizes.

Decreased doubling time normalizes the division length of cells. Doubling time becomes progressively shorter for cells with increased birth length (Fig. 6D). Large newborn cells divide exceedingly rapidly with a doubling time as low as 50 min. Separating doubling time into elongation and septation time shows that the dominant source of regulation occurs in the elongation time (Fig. 6E–F). Elongation time varies over 3.4-fold while septation time only varies by 1.8-fold over the observed length distributions. Septation time is relatively constant for large birth lengths, but is slightly increased for cells with normal birth lengths but small division lengths. Elongation rate also varies only slightly (∼2 fold). Cells dividing at larger sizes exhibit faster elongation rates, which appear to be carried over into the next generation, since cells born with larger sizes also have higher elongation rates. This trend would push cells towards larger sizes and thus is likely counteracted by the reduction in elongation times, which dominates the system.

Comparing the doubling times for sisters and division length of the mother shows that a direct link exists between mother cell length and daughter cell doubling times, as stipulated above (Fig. 7A). Mothers dividing at large size give rise to large daughters that divide again rapidly (short doubling times). Doubling times are also well correlated between the two daughters, with the noise in doubling times increasing the longer the cells require to divide. Cells thus accrue stochastic differences during cell division (cellular insults, DNA damage, etc.) that leads to rapid de-synchronization in doubling times over multiple generations. Due to the inherent link between birth length and doubling times it is not surprising that doubling times of daughters are correlated, since daughters are of similar size. Elongation rates are also correlated between daughters and also correlate with mother division length with larger cells having higher elongation rates. (Fig. 7B).

## Discussion

We developed a microfluidic microchemostat array for the long-term culturing and interrogation of continuously growing *S. pombe* cultures. A growing number of devices are becoming available for microfluidic culturing of *S. cerevisiae* and *E. coli*, but only a few methods have thus far been applied to *S. pombe*, despite its importance as one of the prime model organisms for cell cycle related studies. On our device we could culture *S. pombe* for as long as a week, and on a regular basis for several days. Device clogging is the primary factor limiting culturing times on microfluidic devices which continuously remove cells from the growth regions. On our device cells are grown at high-densities in the individual michrochemostats and are then eluted from the device once cells are pushed into the medium supply channel. If necessary our platform also allows for the implementation of an active cleaning process, in which the michrochemostat chambers can be isolated from the medium supply channels using valves. The medium supply channels can then be cleaned with detergent and/or cell lysis solutions to remove any cellular clusters that may have formed [29]. Such an operating mode could allow culturing of *S. pombe* for up to several weeks. To enable single cell analysis, we ensured that S. pombe grows in well-defined monolayers. We furthermore added ‘highways’ to our michrochemostat growth chambers to guide the cells down a defined path as they transition through the growth chamber. Both aspects, perfect monolayer growth and ‘highways’, make consequent image processing, cell segmentation, tracking, and lineage generation easier. To achieve these image processing tasks we wrote an image processing pipeline capable of returning precision data on cell division timing, growth rates, and cellular morphology.

With the current combination of microfluidic hardware and image processing software we showed that we could analyze over 100000 division events in a single experiment, and follow thousands of cells over 7 generations (Fig. 3B). These numbers should allow the precise analysis of growth phenotypes, which may be particularly useful in the precision analysis of genetic interactions. We have previously shown that over a thousand *S. cerevisiae* strains can be arrayed into individual michrochemostat arrays enabling the high-throughput analysis of cellular phenotypes [25], [30]. A similar approach should also be applicable to *S. pombe* cells since throughput would need to be increased if a large-scale genetic interaction analysis were to be performed. Nonetheless even the current platform can be programmed manually with 8 different *S. pombe* strains permitting the low-throughput analysis of a few dozen strains. The platform is compatible with fluorescent microscopy, making it a suitable platform for the precise investigation of the relationship between intrinsic cellular components that can be fluorescently tagged such as proteins [31], mRNA [32], and metabolites [33]. Combining our platform with these currently available imaging approaches would permit the investigation of how information in noisy cellular environments is transmitted from one generation to the next, and may help establish the mechanism of how *S. pombe* achieves and maintains size homeostasis [34]–[38].

## Methods

### Cell Culture and Strains

Strain WT 972 h- was used in all experiments. Cells were grown overnight at 30°C in 3 ml YES (Difco) 2% glucose with shaking in classical batch culture prior to loading onto the chip. On the device cells were also grown in YES 2% glucose.

### Microfluidic Device

The chip comprises 8 rows of 15 chambers for a total of 120 chambers (Fig. 1A). Each row can be individually loaded with a different strain or perfused with a different medium. Automated medium switches can also be performed. The time resolution is 2.5 minutes if all 120 chambers are imaged. Higher time-resolutions are possible if only a subset of the device is imaged.

Each growth chamber is controlled by 2 valves and can be divided into three zones (Fig. 1B). The first is a sieve region (B2), which allows medium to diffuse into the chamber while preventing cells from escaping. The main section of each growth chamber is the region where cells are cultivated (B4), which is divided by ‘highways’ to provide structural support to the growth chamber and to constrain and guide cell movement. By constraining cell movement, the chamber highways simplify subsequent cell segmentation and tracking. The growth chamber is covered by a micromechanical membrane (B3). Actuating this membrane at low pressure ensures monolayer growth, which is of utmost importance for high-quality imaging and analysis. The last section of the chamber is the exit channel at the chamber outlet. The exit channel can be opened or closed by a chamber valve (B5). The chamber in turn is flanked by two medium channels (B1,6) which can be opened or closed on either side by additional valves. The channel near the sieves (B1) is always free of cells while the channel connected to the chamber outlet (B6) elutes cells exiting from each chamber.

### Mold and Chip Fabrication

Mold fabrication was performed in the clean room facility of EPFL (CMI). Chip designs were drawn using Clewin (WieWeb). The flow wafer consists of 3 successive photoresist layers of different heights. First, a layer of 1040 SU-8 (Gersteltec) was spin coated on a silicon wafer to 1.5 ∼ μm to generate the microfluidic sieves. On this was spin coated a layer of 1050 SU-8 2.5 μm to generate the growth chambers. Each layer was exposed separately. The resulting wafer containing both exposed SU-8 layers was developed twice for 10 minutes in 2-methoxy-1-methylethyl acetate (PGMA). The medium supply channels were generated with a single layer of AZ9260 14 μm. The wafer was then processed at 180°C for 2 hours to round the AZ9260 channels. The control layer is made of a single layer of 1050 SU-8 20 μm exposed and developed using standard techniques. The control layer mask is scaled to 101.5% in order to compensate for PDMS shrinkage during the curing process.

Microfluidic devices were fabricated from polydimethylsiloxane (PDMS) (SYLGARD 184) using standard soft lithography techniques [26]. The control layer was poured at a 5∶1 ratio PDMS and the wafer was degassed for 15 minutes in a vacuum chamber, whereas the flow layer used a 20∶1 PDMS ratio spun onto the flow layer mold at 2600 rpm and cured without a degassing step. Both layers were cured at 80°C for 30 minutes. The control was then cut into 4 devices, and holes were punched to access the control lines. The control layer was then aligned to the flow layer and baked for at least 1 h 30 at 80°C. The chips were then cut, holes were punched for accessing the flow layer and the chips were bonded to glass cover-slips (VWR) at 80°C for 2 hours.

### Chip Operation and Experimental Design

All control lines were primed with water prior to loading the flow layer with medium, except for the chamber outlet valve, which was primed later on. The chip was loaded with medium and cells were seeded in each row. Fig. S3 explains in detail the chip loading procedure. Once the chip was loaded, cells were incubated on-chip overnight in order to ensure normal growth in the new microenvironment. Cells can be theoretically grow on-chip for an unlimited amount of time as excess cells are continuously washed away. In order to prevent clogging, the medium supply channels were purged every 20 minutes by increasing medium flow rate for 5 seconds in each row individually (Fig. S4). This sequence was performed while the top feeding lines of each row were closed.

The device was imaged on a Nikon Ti-E Eclipse and a 40X SPlan Fluor ELWD objective. The microscope was enclosed in a temperature controlled environment (Life Imaging Services). Images were acquired with a Pike F145C IRF16 (Allied Vision Technologies GmbH). All microscope automation, microfluidic device actuation, and imaging were performed with a custom written visual basic program.

All experiments were conducted with the same initial conditions. Cells were imaged for 10–12 hours at 30°C in YES + 2% glucose with 3 psi pressure driving the medium flow. After 10–12 hours we shifted to a new temperature. Temperature was continuously recorded by a thermocouple embedded in a PDMS block with the same dimensions as the microfluidic chip and placed next to the device in order to give an accurate temperature reading. The recorded temperature fluctuations were on the order of ±0.1°C.

### Image Processing and Cell Tracking

The analysis pipeline is described in Figure 2A and Movie S2 gives a graphical representation of the results of the image processing and cell tracking algorithms. First, each image was split into 5 lanes as defined by the microfluidic ’highway’ structures. By imaging slightly out of the focal plane, the septum appeared as a bright white line, whereas the cell contour was black enabling efficient cell segmentation. Each lane was individually segmented based on thresholding and outlining. Cell contours and textures were extracted, and these parameters were used to remove all non-cell features that were segmented in the process (Fig. 2B). Those parameters were also used to sort contours into three categories: i) single cells, ii) features that are too large (>20 pixels (4.6 μm), and iii) features that are too long (>13 μm). The first category was retained directly whereas the last two categories were further refined to reach single cell criteria.

Tracking and lineage reconstruction was performed in 4 successive phases: i) frame by frame cell matching, ii) matrix reconstruction, iii) division detection, and iv) lineage reconstruction. The first phase matched cells frame by frame. To optimize computational speed we first applied a local tracker, which, if it failed due to large cell displacement, was substituted by a global tracker.

Local alignment (Fig. 2C) was applied to all neighboring cells with the following score function:
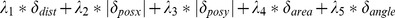
(1)

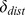
 is the position difference between the center of mass of each boundary. 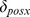 or distx, is the difference on the x axis only, 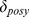 or disty the difference on the y axis, 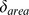 is the difference in area between the 2 cells and finally 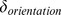 is the angular difference.



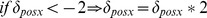
(2)

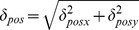
(3)


All cells from frame n were scored to cells in frame n+1. Cells that moved further than a single cell length (50 pixels or 11.52 μm) were scored as 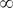 and removed from the process. Candidate pairs with the smallest score are selected if: i) the score is below an arbitrary threshold (65), ii) the cells overlap, iii) and their area doesn’t differ by more than 40%. Pairs that fail to satisfy all three conditions are treated separately. Tracking failure can arise from a split contour (cell division), small local movements, rotations, or low-quality segmentation. Cells for which no satisfactory solution was found were left unpaired to avoid propagating incorrect pairs.

If this local pairing left too many cells unpaired (>30), or the average displacement was bigger than an arbitrary threshold, results were cleared and a frame-by-frame adaptation of the global assignment process proposed by [39] was applied (Fig. 2D). This algorithm minimized the global score of all possible pairings between two frames. Each pair was scored according to the following function.
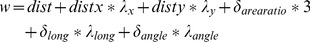
(4)

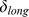
 is the length difference and 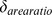 is the difference of area divided by the maximum area of the two cells.

For every lane in a chamber, cells close to the sieve move slower than cells near the outlet. Therefore, we impose minimal displacement constraints on cells depending on their position. Cell pairs failing to achieve this displacement are discarded by the algorithm. If too few cells are paired and the average displacement of paired cells is above 8 pixels (1.85 μm), we reapplied the tracker with a larger minimal displacement. Unpaired cells were then checked as for the local tracker.

In the second phase we constructed a matrix of all paired cells for every frame as defined by both trackers. We detected divisions in the third phase. We generate a lengthwise intensity profile for each cell and convolve it with a Mexican hat function (Fig. S1). Tracking the convolved maximum€s position and intensity over time allows us to detect the evolution of the septum leading to cell division (Fig. S2). This defined cell division events together with cell size information. To compensate for lost cell pairs, we re-seeded untracked cells every 50 frames.

In the fourth phase we reconstruct lineages using cell division information. Cell lineages contain information on individual cell profiles, doubling time, and length at birth. By detecting the time at which growth ceases, we define a plateau, which is the division length. A linear fit of the growth prior to the plateau allows us to obtain the elongation rate.

We estimated the accuracy of our automated analysis by comparing the results of an automated analysis with a manual analysis of the tracked cells from 500 frames. 621 divisions were observed manually and 614 by the software. 7 divisions were missed, among them 2 were reseeded automatically. The remaining 5 errors were either filtered downstream (2) or were lost over the tracking process later on (3). The more critical errors were, 2 divisions that were called too early (1 was filtered out, 1 was lost), 8 cells were wrongly assigned but automatically corrected while tracking and 26 wrongly assigned cells (twice the same cell) but were not automatically corrected. Out of those 26 wrong assignments, 18 were filtered out and 7 were lost in the following steps of the tracking process. In the end only one mistake was actually retained in the results. In summary, out of 614 observed divisions, we obtained 342 paired cells, among them 195 were generation 1, 110 generation 2, and 37 generation 3. After the filtering steps 268 divisions remained. Thus, our algorithm made one error out of 268 divisions, or 0.4%.

## Supporting Information

Figure S1 **Convolution and septum evolution example: Explanation of the progressive appearance and disappearance of the septum.** The first box is the image of a cell, the second represents the raw intensity profile, the third box is the output of the convolution of the raw intensity with the mexican hat filter (Panel A top left inset). This allows the precise detection of the presence or the absence of a septum. A) Is the cell before the septum appearance. B) Shows the beginning of the septum appearance, 16 minutes after panel A). C) The septum reaches peak intensity, 30 minutes after A). D) is 2 minutes after the maximum intensity was reached, the septum quickly disappears. E) 5 seconds after D). F) The septum completely disappeared 1 minute later, the cell is divided and the convolved signal is flat. G) 1 minute later the signal nearly goes the opposite way with a low intensity surrounded by the brighter border of the two cells. Within a 4 minutes time interval, the septum goes from a bright signal to its complete disappearance.
(TIF)

Figure S2 **Single-cell length and intensity profile evolution.** The figure represent the evolution of a given single-cell with its length over time in A), the evolution of the profile intensity in B) and the real images from which the information is extracted in C). A) shows the profile of a single-cell from its birth until the following division, red crosses mark the division time, as explained in the main text the slightly late division call is compensated by the same late call in the previous generation. The long size at the beginning of the length profile is induced by the way the measurements are performed, it basically represents the measurement of both newborn cells that are still linked together in the image while they are split in reality as shown in panel C). Panel B) shows the evolution of the position and value of the maximum of the filtered intensity profile as explained in the main text. We can observe the clear pattern of the appearance of the septum (around half of the growth plateau) and the fast fall afterwards when the cell divides. Panel C) shows the measured cell over time.
(TIF)

Figure S3 **Chip loading procedure.** The valve of the cell free channel is closed on the outlet side while the inlet side remains open. The opposite scheme is applied on the cell excess channel. The outlet valve of the row that is going to be loaded is also opened as well as one medium waste channel on the inlet side. 500 microliters of overnight grown cell culture are introduced in a tygon tube using a syringe. A small volume of liquid is pushed out of the tube such that it makes a small bubble at the end of the metal pin. The tube is then connected on the outlet access hole and is pressurized by removing the syringe and connecting it to the air supply. The pressure was set to 2 psi for cell loading. Cells are then flown within the chambers until a fifth of the space is filled with cells (100–200 cells). The outlet valve of the currently loaded row is closed before the outlet valve of the following row is open to prevent any back-flow in the cell free channel. All the rows are sequentially loaded the same way. The two subsets can be done in parallel as their medium inlets are independent. This loading procedure is done without pressure in the button valve to help cells reach the sieve area. At the end of the loading procedure, all outlet valves are opened, as well as the medium channel. A small pressure is applied in the button valve (1.3 psi) and the washing procedure is activated every 20 minutes. Red crosses represent closed valves while red arrows are opened ones.
(TIF)

Figure S4 **Mechanical washing.** Mechanical washing was activated every 20 minutes to prevent cell accumulation in the device. The flow is increased by first closing the cell free channel for 36 seconds, each row is then sequentially closed for 5 seconds. The washing procedure takes in total 56 seconds. Red crosses represent closed valves while red arrows are opened ones.
(TIF)

Movie S1 ***S. pombe*** **growth on chip.** This movie shows S. pombe growing on-chip in standard Yes medium and an acquisition period of 45 seconds and a total duration of 18 hours.
(MOV)

Movie S2 **Graphical representation of the cell tracking algorithm.** Green outlines are the cell contours in the actual frame and blue outlines show the cell€s position in the previous frame. Pink outlines indicate recently divided cells and red crosses mark the new pole of the cell. White cells are newly seeded cells.
(MOV)
