# Supplementary figures and images for: Long-Term Single Cell Analysis of S. pombe on a Microfluidic Microchemostat Array
Source: PLoS One. 2014 Apr 7;9(4):e93466. doi: 10.1371/journal.pone.0093466 (PMC3977849; doi:10.1371/journal.pone.0093466)

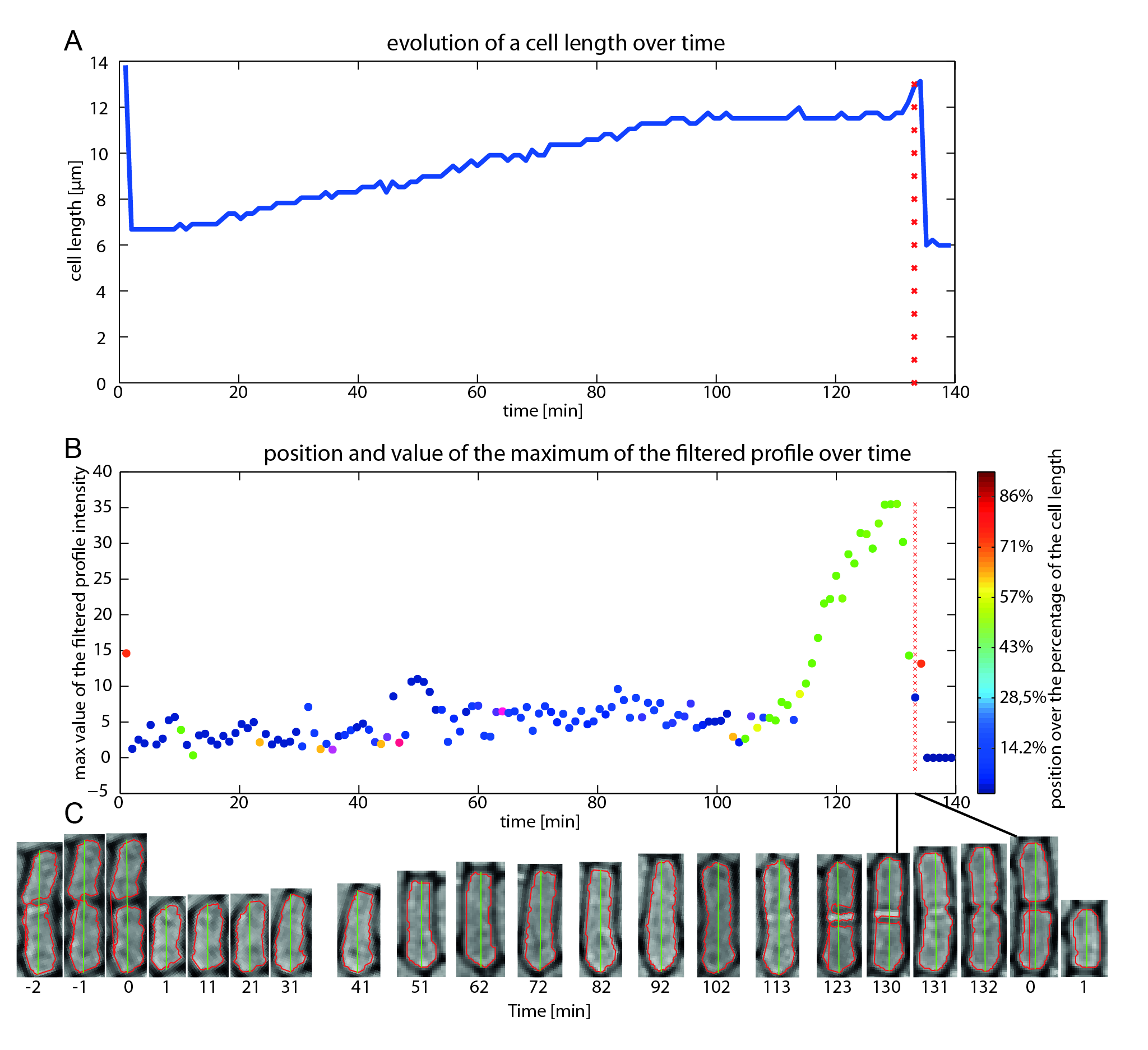

Supplement: Figure S2 — Single-cell length and intensity profile evolution. The figure represent the evolution of a given single-cell with its length over time in A), the evolution of the profile intensity in B) and the real images from which the information is extracted in C). A) shows the profile of a single-cell from its birth until the following division, red crosses mark the division time, as explained in the main text the slightly late division call is compensated by the same late call in the previous generation. The long size at the beginning of the length profile is induced by the way the measurements are performed, it basically represents the measurement of both newborn cells that are still linked together in the image while they are split in reality as shown in panel C). Panel B) shows the evolution of the position and value of the maximum of the filtered intensity profile as explained in the main text. We can observe the clear pattern of the appearance of the septum (around half of the growth plateau) and the fast fall afterwards when the cell divides. Panel C) shows the measured cell over time. (TIF) [file pone.0093466.s002.tif]

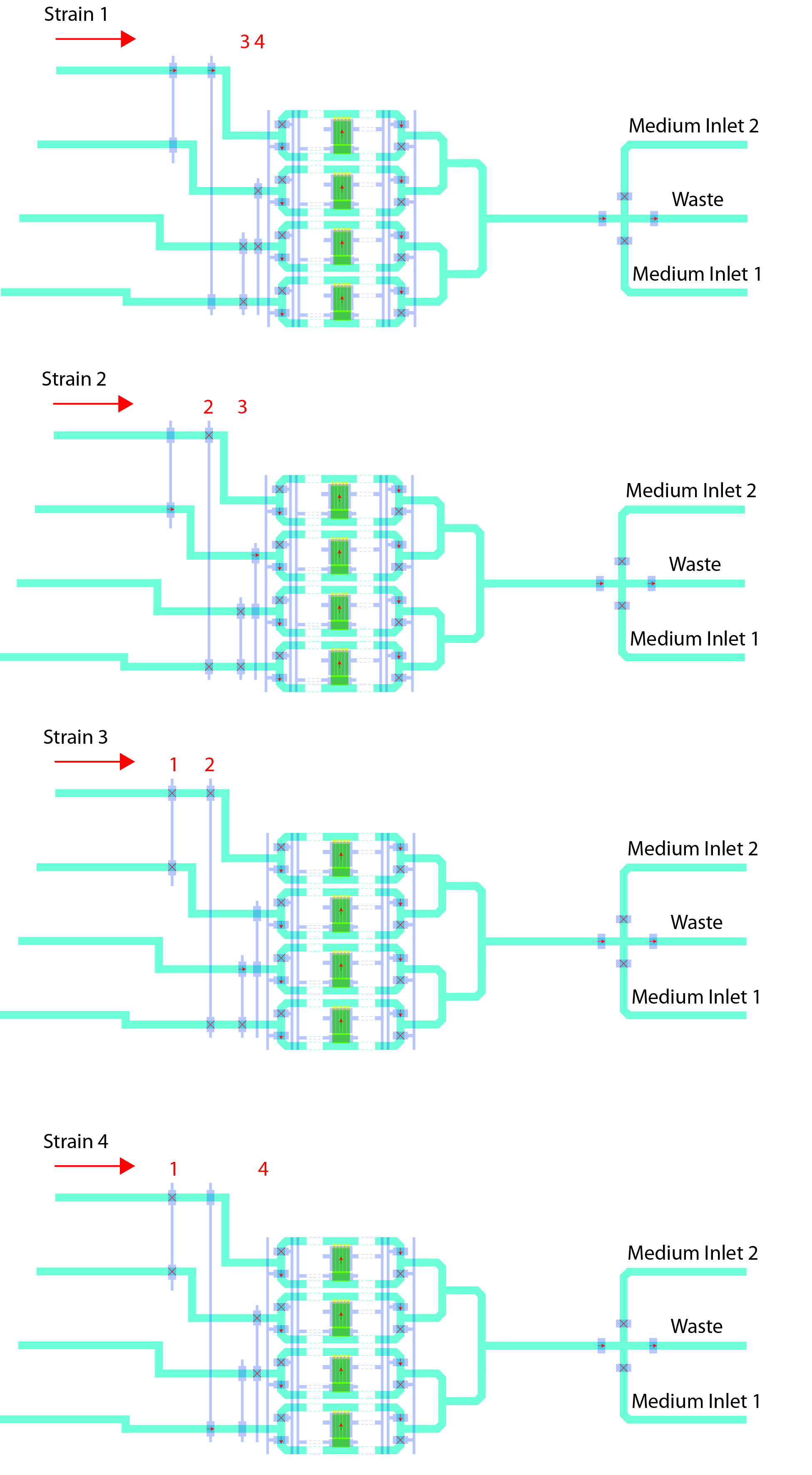

Supplement: Figure S3 — Chip loading procedure. The valve of the cell free channel is closed on the outlet side while the inlet side remains open. The opposite scheme is applied on the cell excess channel. The outlet valve of the row that is going to be loaded is also opened as well as one medium waste channel on the inlet side. 500 microliters of overnight grown cell culture are introduced in a tygon tube using a syringe. A small volume of liquid is pushed out of the tube such that it makes a small bubble at the end of the metal pin. The tube is then connected on the outlet access hole and is pressurized by removing the syringe and connecting it to the air supply. The pressure was set to 2 psi for cell loading. Cells are then flown within the chambers until a fifth of the space is filled with cells (100–200 cells). The outlet valve of the currently loaded row is closed before the outlet valve of the following row is open to prevent any back-flow in the cell free channel. All the rows are sequentially loaded the same way. The two subsets can be done in parallel as their medium inlets are independent. This loading procedure is done without pressure in the button valve to help cells reach the sieve area. At the end of the loading procedure, all outlet valves are opened, as well as the medium channel. A small pressure is applied in the button valve (1.3 psi) and the washing procedure is activated every 20 minutes. Red crosses represent closed valves while red arrows are opened ones. (TIF) [file pone.0093466.s003.tif]

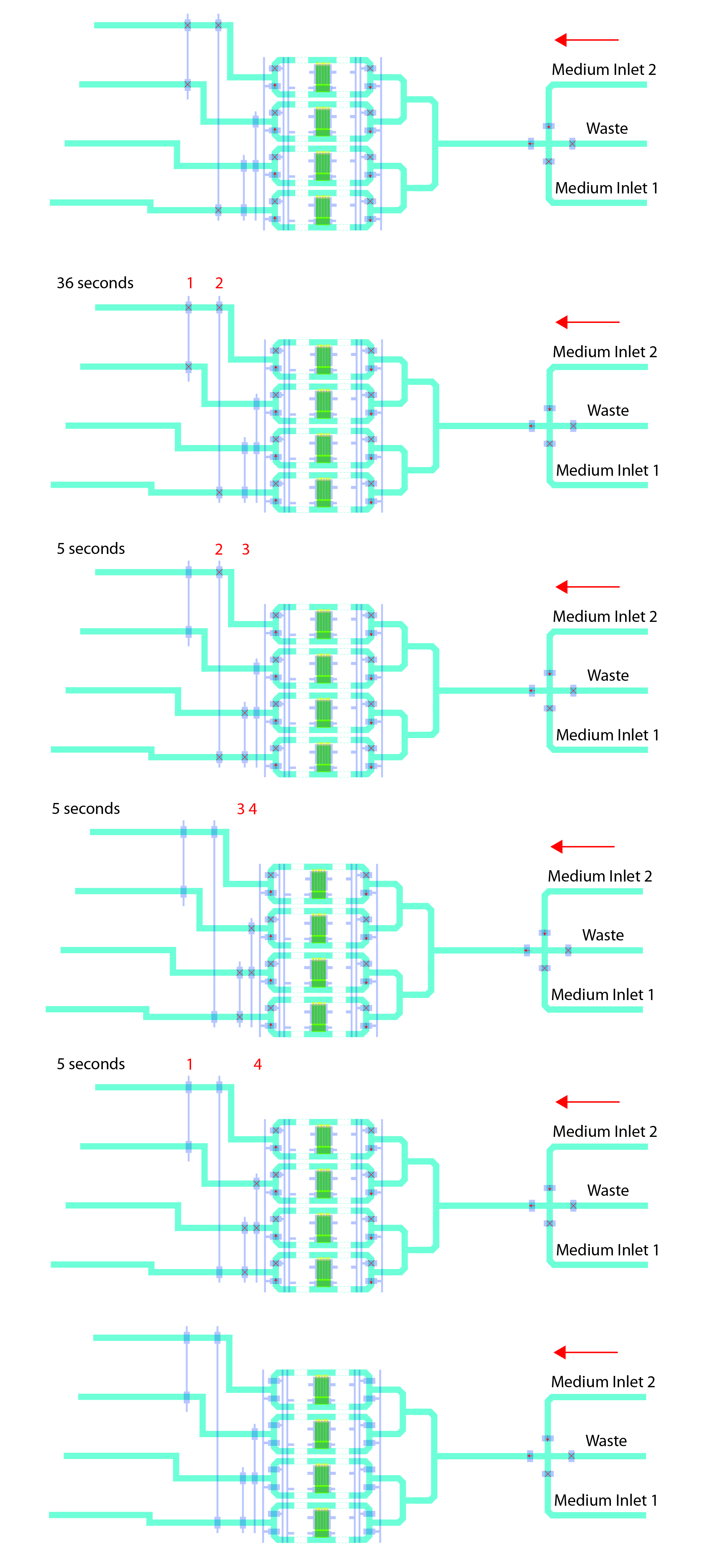

Supplement: Figure S4 — Mechanical washing. Mechanical washing was activated every 20 minutes to prevent cell accumulation in the device. The flow is increased by first closing the cell free channel for 36 seconds, each row is then sequentially closed for 5 seconds. The washing procedure takes in total 56 seconds. Red crosses represent closed valves while red arrows are opened ones. (TIF) [file pone.0093466.s004.tif]
